# Taxonomy of bacterial fish pathogens

**DOI:** 10.1186/1297-9716-42-20

**Published:** 2011-02-02

**Authors:** Brian Austin

**Affiliations:** 1 Institute of Aquaculture, Pathfoot Building, University of Stirling, Stirling FK9 4LA, Scotland, UK

## Abstract

Bacterial taxonomy has progressed from reliance on highly artificial culture-dependent techniques involving the study of phenotype (including morphological, biochemical and physiological data) to the modern applications of molecular biology, most recently 16S rRNA gene sequencing, which gives an insight into evolutionary pathways (= phylogenetics). The latter is applicable to culture-independent approaches, and has led directly to the recognition of new uncultured bacterial groups, i.e. "*Candidatus*", which have been associated as the cause of some fish diseases, including rainbow trout summer enteritic syndrome. One immediate benefit is that 16S rRNA gene sequencing has led to increased confidence in the accuracy of names allocated to bacterial pathogens. This is in marked contrast to the previous dominance of phenotyping, and identifications, which have been subsequently challenged in the light of 16S rRNA gene sequencing. To date, there has been some fluidity over the names of bacterial fish pathogens, with some, for example *Vibrio anguillarum*, being divided into two separate entities *(V. anguillarum *and *V. ordalii)*. Others have been combined, for example *V. carchariae, V. harveyi *and *V. trachuri *as *V. harveyi*. Confusion may result with some organisms recognized by more than one name; *V. anguillarum *was reclassified as *Beneckea *and *Listonella*, with *Vibrio *and *Listonella *persisting in the scientific literature. Notwithstanding, modern methods have permitted real progress in the understanding of the taxonomic relationships of many bacterial fish pathogens.

## Introduction

### "What's in a name?" (William Shakespeare; Romeo and Juliet)

The Swedish botanist Carl Linnaeus (1707-1778), who was also known as Carolus Linnaeus and Carl von Linné, is undoubtedly the Father of Taxonomy, and was responsible for developing a system for naming and ranking living organisms. His lasting contribution was the development of a simplified naming system in Latin with consistency across all living organisms, i.e. the binomial system, in which each organism has a unique two-word name - incorporating genus and species. A simplistic view is that Linnaeus made order out of chaos. Yet, for Linnaeus and his contemporaries, the process was comparatively easy, and involved only large organisms, which were clearly visible to the naked eye (= macro-organisms) and easily seen morphological characteristics (= a category of phenotypic characters). Thus, these early classifications (= the process of arranging organisms into groups) were based on limited but easily visible data, and the outcomes were largely obvious, for example a dog is notably different from a horse and would therefore belong in separate species.

The founding father of microbiology, the Dutch textile merchant and lens maker, Antonie van Leeuwenhoek (1632-1723), observed small organisms initially from the proximity of his teeth (= bacteria and protozoa?), and these entities were termed "animalcules", which he wrote about in a letter to the Royal Society of London in 1676. His careful illustrations suggested morphological variation between the cells. Yet, another two centuries were to pass before serious attempts at naming and ordering bacteria started. Thus, bacterial taxonomy has progressed from the simplistic approach involving a small number of readily observable characteristics, such as morphology as deduced from observation using light microscopes, to the modern applications of molecular biology. With improvements in knowledge, there have been refinements in taxonomic processes and an increase in reliability. It should be remembered that taxonomy (= the theory of classification, nomenclature and identification) is a man-made process, i.e. the organisms included in any classification have not chosen to be placed in the groups that have been created by human beings. Nevertheless if done properly, taxonomy has value in:

• Understanding biodiversity, namely the range of organisms in a given habitat

• Communication between scientists, thus enabling exchange of information about similar organisms

• Cataloguing information - the name is the key to a catalogue of information about the organism

• Enabling identification, such that new isolates may be readily and reliably identified

• Providing an insight into evolutionary pathways (= phylogenetics).

To be effective, taxonomy should be

- based on a high information content

- reproducible, and

- stable,

otherwise confusion will surely result.

Since the start of bacterial taxonomic processes in the nineteenth century, there has been a progression in the type of information used in the procedure. It may be argued that early bacteriologists had considerable taxonomic insight judging from the conclusions reached from the comparatively simple data that were available. However, taxonomy is a dynamic science, with new developments/methods being incorporated into processes including the descriptions of bacterial species. Since the 1950 s, bacterial taxonomy has evolved rationally, encompassing numerical methods [1,2], chemotaxonomy (e.g. [3,4]), and molecular techniques [5]. Taxonomy has progressed from a highly artificial process involving limited amounts of phenotypic data to the recognition of more natural relationships between organisms, based on comparatively large amounts of varied and reliable data covering multiple aspects of the biology of an organism, and including phenotypic, chemotaxonomic, genotypic and phylogenetic date, i.e. a polyphasic approach. However, the current dominance of 16S rRNA gene sequencing although revolutionising some aspects of bacterial classification needs to be treated cautiously as overreliance on the approach may lead to erroneous conclusions [5]. Nevertheless, it is apparent that sequencing methods are instrumental with the explosion of new species names, which have greeted the arrival of the twenty-first century. Whereas, the information content of many of the new species descriptions is generally high, an unwelcome trend is that many new taxa (= taxonomic groups) are described solely after the study of only single strains. Therefore, the diversity/variability within the new taxon cannot be adequately assessed. Also, it is impossible to determine whether a single strain is effectively an outlier or a median representative of the group (in future years, will it be regarded as typical or atypical of the group?). However, taxonomy is often ignored by many microbiologists in other specialisms, and there may well be concern that basic principles could be forgotten, e.g. is the purity and authenticity of cultures always verified before use? Where culturing is not possible, there is the possibility of analyzing the nucleic acids, determining species composition, and even proposing new taxa, i.e. by the use of culture-independent approaches.

## Bacterial fish pathogens

There has been a steady increase in the numbers of bacterial species associated with fish diseases, with new pathogens regularly recognised in the scientific literature [6]. However, the names of many bacterial fish pathogens have been subjected to taxonomic change over time, with some species split, for example *Vibrio anguillarum *biotype 2 becoming re-classified as a separate species *V. ordalii *[7,8]. In other cases, different nomenspecies have been combined, for example *V. carchariae, V. harveyi *and *V. trachuri *into *V. harveyi*, which had precedence because it was the first name to be proposed, albeit as the luminous *Achromobacter harveyi *[9-11]. The oldest known fish pathogen, *V. anguillarum*, has undergone name changes to *Beneckea *[12] and *Listonella *[13]; neither of which was widely accepted. However, *Listonella *remains a valid name and is mentioned in the current edition of Bergey's Manual of Systematic Bacteriology, and *Beneckea *has been consigned to the history books. A positive aspect of sequencing methods is that there has been a progression towards the Orwellian notion of "Order out of Chaos" even if scientists do not always appreciate the significance of the data.

## Isolation of fish pathogens: the culture-dependent approach

With the rapid development in molecular biology, it is not always necessary to culture an organism in order to enable its study, including the allocation of a species name. Thus, the concept of culture-independent techniques was developed and refined. Sensitivity and specificity increased, but without culturing there was an inability to carry out associated studies, such as the determination of pathogenicity factors. The attraction of culture-dependent approaches is that a pure culture may be obtained and deposited in culture collections as reference material for use by others. This raises a concern about the usefulness of cultures. An assumption is made that pathological material may be used for the recovery of a pure culture of the aetiological agent. This will depend on using appropriate media and incubation conditions, and assumes that the organism is in a form that may be cultured and that the microbiologist picks the "correct" colony. If mixed growth occurs or if the pathogen is largely overgrown by opportunists/secondary invaders/saprophytes, then there is concern that the actual pathogen will be missed. In addition, infections resulting from two or more organisms working synergistically will undoubtedly be mis-diagnosed if the diagnostician chooses only one culture for study. However, there are only a comparatively few indications of disease resulting from multiple species, such as *Aeromonas hydrophila *with *A. salmonicida *[6]. It is speculative how many diagnoses (if any) are made of contaminants rather than the actual pathogen. Moreover, it is surprising that only two species of anaerobic bacteria, namely *Clostridium botulinum *and *Eubacterium tarantellae*, have been implicated as fish pathogens [6]. Of course, this could reflect the general lack of use of appropriate anaerobic procedures by microbiologists rather than the absence of anaerobic pathogens.

## Approaches to characterization

### Phenotype

Traditionally, bacteria were characterized phenotypically, and undoubtedly for some groups, e.g. the Enterobacteriaceae, a wealth of knowledge emerged particularly from the 1950's onwards. Currently, emphasis on phenotype has declined with a concomitant move towards molecular-based approaches. Nevertheless, phenotypic data have a role in polyphasic studies whereby many facets of the biology of an organism are studied [14]. Phenotyping leads the way with diagnoses worldwide; emphasis often being placed on commercial kits and the use of manufacturer's probabilistic databases to achieve an acceptable identification. Although the approach has standardized diagnoses, the weakness is that most identification systems have been developed for medically important bacteria that grow within 24-48 h at 35-37°C. Consequently, the reliability of these kits for use with fish pathogens which need lower incubation temperatures for longer periods needs to be questioned [6,15]. In particular, the API 20E system includes the use of sugar fermentation reactions, which may be influenced by the presence of plasmids [6]. Moreover, there may be confusion over the interpretation of the profiles. For example, some of the profiles of *A. hydrophila *are similar to those of *A. allosaccharophila *and *A. sobria; Yersinia ruckeri *may be confused with *Hafnia alvei; *moreover *Tenacibaculum maritimum *and *Pseudomonas anguilliseptica *are indistinguishable by API 20E [6]. Problems may result when data from rapid commercial kits are used in conjunction with conventional diagnostic schemes, which have been developed for traditional and often laborious phenotypic characters. Also, some of the traditional tests, e.g. the Voges Proskauer reaction, are not noted for their reproducibility and may introduce errors into the taxonomic process and lead to mis-identification [16].

Undoubtedly, selective media have proved useful for the recovery of some fish pathogens, with an example including selective kidney disease medium (SKDM) for *Renibacterium salmoninarum *[17]. However, selective media are only available for a minority of all fish pathogens, therefore recovery is dependent on more general culturing methods. Specially developed diagnostic procedures have aided identification of some group, e.g. the glucose motility deep cultures have benefitted the recovery and identification of *V. anguillarum *[18].

### Immunological methods

The development and availability of standardized immunological (antibodies and kits) reagents have improved diagnoses considerably [6,19,20], and enhanced the reliability of methods for the detection of pathogens, including *Mycobacterium *spp., *Photobacterium damselae *subsp. *piscicida *[21]*, Piscirickettsia salmonis *[22], *R. salmoninarum *[23] and *Streptococcus iniae *[24]. Tentative diagnoses, including of asymptomatic infections, may result from use of monospecific polyclonal or monoclonal antibodies in a range of antibody-based procedures, including the indirect fluorescent antibody test (iFAT), whole-cell (slide) agglutination, precipitin reactions, complement fixation, immunodiffusion, antibody-coated latex particles, co-agglutination using antibody-coated staphylococcal cells, passive haemagglutination, immuno-India ink technique (Geck) or enzyme linked immunosorbent assay (ELISA; reviewed by [25]), the latter of which may also be used for serology, i.e. detecting antibodies in the host to specific pathogens [26]. Antibody-based methods are used effectively for detecting exposure to fish viruses, such as Koi herpes virus [27], but bacterial pathogens pose a more complicated picture with cross reactivities likely unless specific known molecules/antigens are used to coat the ELISA plates rather than whole pathogens [19]. Techniques are often sensitive, specific, rapid and reliable, and in some cases may be used in the field [6]. This is in marked contrast to molecular biology, which may be much slower and relies on specialist, well equipped laboratories.

### Chemotaxonomy

Chemotaxonomy involves the investigation of chemical constituents of bacteria, and is particularly useful for the study of Gram-positive bacteria. The molecules studied include fatty acids, polar lipids, lipopolysaccharide (nature of the chain length of the fatty acid and the sugar in the Lipid A moiety; [28]), menaquinones, naphthoquinones, ubiquinones, mycolic acids, peptidoglycan, polyamines, teichoic and teichuronic acids and isoprenoid quinones [29]. Mycolic acids, which are useful taxonomic markers, are present in Gram-positive bacteria with high G+C ratios of their DNA [4], and have been reported for a range of fish pathogens, including *Mycobacterium chelonei *subsp. *piscarium *[3] and *M. shottsii *[30]. The length of the mycolate side chain has been correlated to 16S rRNA gene sequence homology [31]. Specific examples for which reliable chemotaxonomic data exist for Gram-positive bacterial fish pathogens are detailed below:

#### Lactococccus piscium

The long chain cellular fatty acids of *Lactococcus piscium *were reported to be straight chain saturated, mono-unsaturated and cyclopropane-ring types. The major acids corresponded to hexadecanoic acid, ∆ 11-octadecanoic acid and ∆ 11-methylenoctadecanoic acid [32].

#### Mycobacterium neoarum

The cell wall chemotype has been given as IVA, with glycolated muramic acids, mycolic acids and MK-9, as the predominant isoprenoid quinone, being present [33].

#### Nocardia

*Nocardia salmonicida *contains LL-diaminopimelic acid (DAP) and glycine but not meso-DAP, arabinose or galactose in the cell wall (i.e. Type I). The major cellular fatty acids are hexdecanoic, octadecanoic, octadecanoic and 10-methyloctadecanoic acid [34].

*N. seriolae *contains *meso-*diaminopimelic acid, arabinose and galactose, indicative of chemotype IVA. Mycolic acids containing 44-58 carbon atoms are present. The cellular fatty acids are dominated by *n-*C_16:0_, *n*-C_16:1 _and *n-*C_18:1_; 10-methyl-C_19:0 _is also present as a major component in some isolates. The predominant isoprenoid quinone is tetrahydrogenated menaquinone with eight isoprene units [34].

#### Renibacterium salmoninarum

Chemotaxonomic traits of *R. salmoninarum *have been highlighted in part because of the comparative difficulty with obtaining conventional phenotypic test results. Thus, the cell wall peptidoglycan was deduced to contain D-alanine, D-glutamic acid, glycine and lysine as the diamino acids [35]. The principal cell wall sugar was glucose, although arabinose, mannose and rhamnose were also present; mycolic acids were absent [36]. Methyl-branched fatty acids form > 92% of the total fatty acid component of the cells, with 12-methyltetradecanoic (anteiso-C_15_), 13-methyldecanoic (iso-C_15_) and 14-methylhexadecanoic (anteiso-C_17_) as the major components. Straight chain fatty acids generally account for 1% of the total fatty acids, and unsaturated fatty acids are not detected at all. Over 81% of the total fatty acids are composed of the lower melting point anteiso acids, which may contribute to membrane fluidity at low temperatures. Unsaturated menaquinones with nine isoprene units are present. All strains contain diphosphatydylglycerol, 2 major and 6 or 7 minor glycolipids and two unidentified minor phospholipids [37].

### Molecular/genetic methods

Molecular/genetic methods involving 16S rRNA gene sequencing [38], reverse transcriptase-sequencing [39,40] and polymerase chain reaction (PCR)-based gene sequencing [41] have been useful additions to the armoury of techniques applicable to bacterial taxonomy [29,31]. DNA hybridization, which is regarded as the "gold standard" for demonstrating the presence or absence of new species, was introduced into bacterial taxonomy during the 1960s (e.g. [42]). Genotypic classification involving sequencing of the 16S and 23S RNA genes (the latter is less popular) is regarded as the definitive standard for determining phylogenetic relationships of bacteria [29,38]. In particular, the genes are regarded as having all the attributes of useful, relevant and stable biological markers being present and homologous in all bacteria. Also, they are not prone to the effects of gene transfer [29]. Yet, the exact homology values have a profound effect on interpretation of the outputs. Thus, homology values of ≤ 98.7% (97% according to [31]) indicates membership of different species, and this correlates well with DNA hybridization results. Yet, occasionally higher homology values may be attributed to distinct species groupings [43]. By themselves, 16S rRNA gene sequences are insufficient to describe a new species, but may be used indicatively and in conjunction with DNA:DNA hybridization [31]. However, sequencing has permitted the recognition of new variants. For example, sequencing revealed a new variant among Israeli isolates of *Streptococcus iniae *[44]. Moreover, 16S rRNA cataloguing has been useful in providing information about the position of species in existing classifications. Thus, small-subunit rRNA sequencing and DNA:DNA hybridization revealed that *Pasteurella piscicida *was related to *Photobacterium damsela *leading to the proposal that the pathogen be re-classified as *Ph. damsela(e) *subsp. *piscicida *[45]. Furthermore, *R. salmoninarum *was deduced to be a member of the actinomycete subdivision, being related to *Arthrobacter, Brevibacterium, Cellulomonas, Jonesia, Micrococcus, Promicromonospora, Stomatococcus *and *Terrabacter *[46,47]. The evolutionary relationship of *R. salmoninarum *to *Arthrobacter *was reinforced as the result of genome sequencing, which suggested that the genome of the former had been reduced significantly since its divergence from a common ancestor [48].

Nucleic acid fingerprinting methods, including amplified fragment-length polymorphism PCR (AFLP), pulsed field gel electrophoresis (PFGE), random amplified polymorphic DNA (RAPD), rep-PCR (repetitive element primed PCR), REP-PCR (repetitive extragenic palindromic-PCR), ERIC-PCR (enterobacterial repetitive intergenic consensus sequences-PCR), BOX-PCR (derived from the boxA element) and ribotyping, provide information at or below the subspecies level [31]. Of these, AFLP and ribotyping are extremely useful and standardized.

It is unfortunate that with the increasing use of molecular methods, the description of bacterial groups has been often met with the use of minimal phenotypic data, which causes problems for diagnostics especially in laboratories, which are not equipped for molecular biology [6]. In these situations where distinguishing phenotypic feature have not been or could not be provided then the species should be referred to as a geno[mo]species. Nevertheless, molecular methods have revolutionized taxonomy, and led to the description of an increasing number of new taxa. The methodologies may be culture-independent, allowing for the study of uncultured organisms but there are issues with genomic fluidity [29]. "*Candidatus*" describes uncultured prokaryotes for which phylogenetic relationships have been determined, and authenticity confirmed by methods such as in situ probing [29].

Sequencing of the 16S rDNA is becoming an accepted procedure for the identification of fish pathogens, for example *V. harveyi *[49] and confirming its synonymy with *V. carchariae *[9,10], and has been instrumental in the recognition of new pathogens, including *Streptococcus dysgalactiae *[50], *S. parauberis *(previously recognised as *S. uberis *genotype II; [51]) and *Vagococcus salmoninarum *[8,52], and confirmed the presence of *Lactococcus garvieae *in Taiwan [53].

DNA:DNA and RNA:DNA hybridization, 16S RNA cataloguing, and 5S and 16S rRNA sequencing techniques have been used with increasing regularity and success. A review of PCR with emphasis on validation of the techniques and problems with diagnosis has been published [54]. PCR has been used successfully to identify hard-to-isolate fish pathogens, such as *Mycobacterium *spp. in sea bass (*Dicentrarchus labrax) *[55] and *M. chelonei *in a cichlid oscar *(Astronotus ocellatus) *[56]. Moreover, PCR has been useful with distinguishing different species from within the same genus, such as *Lactococcus garvieae *from *L. lactis *[57], from related genera, i.e. *L. garvieae, S. difficilis, S. iniae *and *S. parauberis *[58], and with an admirable level of specificity [59].

The sensitivity of PCR is clearly a positive attribute particularly with slow growing and/or nutritionally fastidious pathogens that are otherwise difficult to study in the laboratory. Of relevance, a PCR was developed [60,61], which detected only 22 cells of *R. salmoninarum; *a sensitivity of 10 cells was reported by others [62]. Similarly, PCR detected only 10^2 ^colony forming units (CFU) of *N. seriolae *in yellowtail [63].

A recent development is multi locus sequence analysis (MLSA), which permits the genotypic examination of micro-organisms by comparison of the sequences of multiple, i.e. 12 or more, house-keeping genes. The benefit of using multiple genes is that the outputs are more informative and less likely to generate results that are distorted by recombination of single loci [29]. The resulting phylogenetic trees are capable of recognizing deeply branching clusters and permit the delineation of groups within a species or genus [64].

### New species of fish pathogens recognized by 16S rRNA sequencing

16S rRNA sequencing has helped the description of fish pathogens where phenotypic characterization alone does not permit their incorporation in classifications. For example, a new disease of Atlantic salmon (*Salmo salar*) was linked to the *Streptobacillus moniliformis *and the fusobacteria group on the basis of sequence homology; biochemical traits did not permit identification [65]. The newly described cause of a mycobacteriosis in Chesapeake Bay (USA) striped bass *(Morone saxatilis) *was equated to a new species, *M. shottsii*, with confirmation by 16S rRNA sequence homology in which the pathogen was linked most closely to *M. marinum *and *M. ulcerans *(similarity = 99.2%) [30]. In one study, *M. gordonae *was identified by 16S rRNA sequencing [66]. Furthermore, phylogenetic analysis based on 16S rRNA gene sequencing together with partial sequences from the 65 kDa heat-shock protein (hsp65) and the beta-subunit of the bacterial RNA polymerase (*rpoB*) genes and the 16S-23S internal transcribed spacer 1 (ITS 1) region named other novel mycobacteria as *M. stomatepiae *and *M. barombii *[67].

During an examination of 16S rRNA sequences, two isolates of motile aeromonads from diseased elvers in Spain were described as a new species, *Aeromonas allosaccharophila *[68], albeit phenotypically heterogeneous [69]. This heterogenicity has caused problems for reliable phenotypic-based diagnoses.

*Francisella *became recognized as the cause of a new disease of Atlantic cod (*Gadus morhua*) in Norway in which the affected fish displayed white granuloma in the viscera and skin. Isolates were recovered, and determined to possess the key phenotypic characters of *Francisella*, viz. non-motile, strictly aerobic Gram-negative intracellular coccobacilli which produced H_2_S from cysteine-containing media [70]. 16S rRNA sequencing revealed a 99.17% homology to *Francisella philomiragia *[71], although a slightly higher value of 99.3% was published [70] with the proposal for a new subspecies, i.e. *Francisella philomiragia *subsp. *noatunensis*, to accommodate the organisms. There was 92.2-99.0% homology with *Francisella philomiragia *housekeeping genes, *groEL, shdA, rpoB, rpoA, pgm *and *atpA*. A DNA:DNA hybridization of 68% was recorded between the fish pathogen and *Francisella philomiragia *[70].

*Pasteurella skyensis *was recovered from diseased Atlantic salmon in Scotland, linked to the family Pasteurellaceae by phenotypic analysis, and elevated to a new species largely as a result of 16S rRNA sequencing that identified the closest neighbour as *Pasteurella phocoenarum *(homology = 97.1%; [72]).

*Piscirickettsia salmonis *was named to accommodate isolates from diseased salmon in Chile, of which LF-89 was studied in detail [73] with 16S rRNA conforming to the gamma subdivision of the Proteobacteria with similarities to the family Rickettsiales, and in particular *Wolbachia persica *(similarity = 86.3%) and *Coxiella burnetii *(similarity = 87.5%) more than to representatives of *Ehrlichia, Rickettsia *or *Rochalimaea *leading to the description of a new genus and species [73]. Other rickettsias not conforming exactly with *Piscirickettia salmonis *have been described. For example, an organism recovered from white sea bass (*Atractascion nobilis*) was reported to have a 96.3-98.7% 16S rDNA homology with *Piscirickettsia salmonis *[74], which was considered by the authors to be too low for a confirmed identity. A Tasmanian isolate from Atlantic salmon was distinct from *Piscirickettsia *in terms of sequence alignment of the 16S rRNA, and for the present regarded as a rickettsial-like organism (RLO; [75]).

*Pseudomonas plecoglossicida*, the causal agent of bacterial ascites of ayu *(Plecoglossus altivelis)*, was described as a new species as a result of 16S rRNA gene sequence analysis confirming distinctiveness from *P. putida *biovar A. DNA:DNA hybridization confirmed the isolates to be a new centre of variation insofar as < 50% homology was recorded with other pseudomonads, including *P. putida *[76].

*Streptococcus phocae *was recognized as a cause of systemic disease in Atlantic salmon farmed in Chile. Phenotypic testing linked the pathogen with the streptococci, notably *Gemella*, but analysis of 16S rRNA genes provided a link to *S. phocae *[77].

*Tenacibaculum soleae *was recovered from diseased sole *(Solea senegalensis) *in Spain, and confirmed as a new species largely on account of 16S rRNA homology values of 94.8-96.7% with other members of the genus [78].

Two groups of bacteria were recovered from Atlantic salmon with winter ulcer disease/syndrome [79], of which one cluster was found to be closest to *Moritella marina *(43% re-association by DNA:DNA hybridization), and was named as *V. viscosus*. By 16S rDNA sequencing, the closest match was with *Moritella *[79] and *M. marina *(99.1% sequence homology) so that the organism was re-classified to *Moritella*, but as a new species, as *M. viscosa *[80], despite the high sequence homology [29]. Separately, 19 Icelandic and one Norwegian isolate from shallow skin lesions on Atlantic salmon, and the type strain of *V. marinus *NCIMB 1144 were identified as *V. marinus *after an examination of phenotypic data and analyses by numerical taxonomy [81]. On the basis of 16S rRNA sequencing, the species was transferred to *Moritella *as *M. marina *[82].

### New and uncultured fish pathogens: "*Candidatus*"

Molecular techniques have permitted the recognition of uncultured pathogens belonging to new groupings for which the name of "*Candidatus*" has been used. "*Candidatus *arthromitus" has been recovered from rainbow trout (*Oncorhynchus mykiss*) with summer enteritic syndrome, which is a gastro-enteritis [83,84]. The organism was observed in histological preparations to which nested polymerase chain reaction was used, with confirmation by sequencing [85]. "*Candidatus *piscichlamydia salmonis" was detected by RT-DGGE in intracellular inclusions, i.e. epitheliocysts, in Atlantic salmon with proliferative gill inflammation [86]. "*Candidatus *clavochlamydia salmonicola" is an intracellular organism, causing epitheliocystis in Atlantic salmon, which was recognized as novel as a result of 16S rRNA sequencing [87].

## Taxonomic developments associated with specific bacterial fish pathogens

From the early literature, a question-mark has hung over the reliability of some bacterial names insofar as there was often negligible evidence to support the use of those names. Concern may also be expressed about the value of studies based on only single isolates where concern about the reasons for choice of the culture may be aired. Some of the controversy surrounding specific diseases/pathogens follows:

### Motile aeromonas septicaemia

*Aeromonas hydrophila *(= *A. formicans *and *A. liquefaciens) *would appear to have worldwide distribution and to be a pathogen, causing motile aeromonas septicaemia, of many species of freshwater fish. Indeed, there are reports of a spread into marine fish, notably ulcer disease of cod [88]. Since its initial recognition in the literature, a wealth of knowledge has been accumulated about many facets of its biology (see [6]). A new variant *A. hydrophila *subsp. *dhakenis*, which was originally recovered from children with diarrhoeae in Bangladesh, was determined to be pathogenic to rainbow trout [89]. However overall, there has been some doubt about the role of *A. hydrophila *as a pathogen, and in some cases it may well be present in fish tissue only as a secondary invader [6]. Moreover with developments in the taxonomy of motile aeromonads [90], the accuracy of some of the early published identifications may be justifiably questioned. Could other motile aeromonads be associated with fish disease and may have been confused previously with *A. hydrophila *[89]?

It is clear that there is phenotypic, serological and genotypic heterogeneity within the descriptions of fish pathogenic *A. hydrophila *[e.g. 91, 92], and other motile aeromonads have been implicated as the aetiological agents of (fish) diseases. Thus, 8 isolates reported as pathogenic to eel in Spain were identified by numerical taxonomy as *A. jandaei *[93,94]. Certainly, the current approach of allocating species names as a result of the examination of 16S rRNA gene sequences has encompassed fish pathogenic motile aeromonads. For example, isolates from diseased fish which were recovered in *Aeromonas *DNA Hybridization Group 2 (= *A. hydrophila) *were equated with a new group, *A. bestiarum *[95]. Subsequently, *A. sobria *(*A. sobria *biovar sobria and *A. veronii *biovar sobria were reported as pathogenic to rainbow trout [89]. Indeed, *A. sobria *has been previously found to have a role as a fish pathogen, with isolates recovered from wild spawning gizzard shad (*Dorosoma cepedianum) *in Maryland, USA during 1987 [15,96]. Also, *A. veronii *has been recovered from Siberian sturgeon (*Acipenser baerii*) with identification of the pathogen resulting from phenotyping and 16S rRNA gene sequencing [97].

#### Aeromonas salmonicida

*Aeromonas salmonicida *is one of the oldest described fish pathogens, being isolated initially from diseased hatchery-maintained brown trout (*Salmo trutta*) in Germany, and named as "Bacillus der Forellenseuche" or bacillus of trout contagious disease. The history of the organism reveals a plethora of synonyms including *Bacillus devorans*, *Bacterium salmonica*, *Bacterium salmonicida*, *Bacillus truttae *and *Bacillus salmonicida *[6]. The 7th edition of *Bergey's Manual of Determinative Bacteriology *(1957) placed the pathogen in the genus *Aeromonas *within the family Pseudomonadaceae [98]. Later, there was a transfer to the family Vibrionaceae and subsequently to its own family, i.e. the Aeromonadaceae [99]. Re-classification was based primarily on phenotyping [100]. Thus in 1953, the first detailed description of the pathogen was published, and from an examination of 10 isolates, it was concluded that *Bacterium salmonicida *was homogeneous in cultural and biochemical characteristics [100]. Numerous studies have addressed the homogeneity of the species (e.g. [101]). The basic description is of an organism, which comprises non-motile (motility and *flaA *and *flaB *flagellar genes have been reported [52,82]), fermentative, Gram-negative rods, which produce a brown water-soluble pigment on tryptone-containing agar, do not grow at 37°C, and produce catalase and oxidase [6]. Cultures have the ability to dissociate into rough, smooth and G-phase (= intermediate) colonies [102]. The pathogen has spread from its dominance in salmonids to cyprinids and marine flatfish [6]. An ongoing issue surrounds the intraspecies structure, i.e. the validity of subspecies *achromogenes, masoucida, pectinolytica, salmonicida *and *smithia*, and the status of so-called atypical isolates.

*A. salmonicida *subsp. *salmonicida*, isolates of which have been obtained almost exclusively from outbreaks of furunculosis in salmonids, is regarded as homogeneous, and is referred to as "typical" [103]; all other isolates are considered as heterogeneous and "atypical" [6]. So called atypical strains may demonstrate weak, slow or non-pigment production [104,105], catalase [106] or oxidase-negativity [e.g. 106, 107], nutritional fastidiousness for blood products [108], slow growth, i.e. ≥ 5 days compared with 1-2 days for typical isolates [106,108], and be pathogenic for fish other than salmonids, e.g. cyprinids (e.g. [106,108,109]) and marine flatfish, namely dab (*Limanda limanda*), plaice (*Pleuronectes platessa*), flounder (*Platichthys flesus*) and turbot (*Scophthalmus maximus*) [110-112], and cause ulceration rather than furunculosis [6,113]. The deviation in characteristics from the typical to atypical isolates has made typing difficult [114-116]. Even 16S rDNA sequencing has not helped with the clustering of atypical forms (e.g. [117]).

Smith [118] recognized heterogeneity in the species description of *A. salmonicida*. She examined six isolates of non-pigmented *A. salmonicida*, which were clustered as Group I in her numerical taxonomy study, for which a separate new species name was proposed, i.e. *A. achromogenes*, but the proposal was not adopted widely. A second non-pigmented group was described by Kimura [119], and named as *A. salmonicida *subsp. *masoucida*. Schubert [120] considered these non-pigmented isolates as subspecies, and coined the names of *A. salmonicida *subsp. *achromogenes *and *A. salmonicida *subsp. *masoucida*, respectively. Pigmented strains (= typical) were classified as *A. salmonicida *subsp. *salmonicida *[120]. The precise relationship of the subspecies has been the subject of detailed discussion. In particular, it was contended that subsp. *achromogenes *and *masoucida *were more closely related to *A. hydrophila *than to *A. salmonicida *[121]. Later, it was mooted that subsp. *masoucida *bridged typical *A. salmonicida *and *A. hydrophila *[122]. Yet, *A. salmonicida *subsp. *masoucida *is non-motile, sensitive to *A. salmonicida *bacteriophages, possesses an antigenic profile specific to *A. salmonicida*, and shares a DNA homology of 103% with *A. salmonicida *[92]. By PCR, a combination of *achromogenes *with *masoucida *could be justified, but this was not substantiated by ribotyping and RAPD analyses [114]. Phenotypic data suggest a case for combining subsp. *masoucida *with *salmonicida*, and subsp. *achromogenes *with *Haemophilus piscium*, which is the causal agent of ulcer disease of trout [123]. Examination of the small subunit rRNA gene sequences revealed 99.9% homology of an authentic strain of *H. piscium *with *A. salmonicida *subsp. *salmonicida *[124]. So far, the comparative uniqueness of subsp. *smithia *has been indicated from several studies (e.g. [114]). The complication is with aberrant strains of *A. salmonicida *from fish species other than salmonids.

DNA homology was used to reveal that all isolates of *A. salmonicida *(including *A. salmonicida *subsp. *masoucida) *were highly related, i.e. 96-106% homology, when hybridized against *A. salmonicida *subsp. *salmonicida *[92]. It was opined that *A. salmonicida *subsp. *masoucida *and some atypical isolates did not warrant separate subspecies status, because they were regarded as variants of other well-recognized groups. Also as a result of genotypic analyses, it was reported that typical and atypical isolates of *A. salmonicida *were very closely related, with minimal divergence [125]. Using DNA:DNA re-association, it was concluded that typical *A. salmonicida *were recovered in a homogeneous group, whereas the atypical representatives were more diverse [126]. From numerical taxonomy and DNA:DNA hybridization, similar conclusions resulted regarding the homogeneity of typical isolates of *A. salmonicida *[101]. However using 16S rRNA sequencing techniques, it was reported that subspecies *achromogenes *and *masoucida *were indistinguishable, and only differed from subspecies *salmonicida *by two bases [68].

The relation of *A. salmonicida *to other aeromonads has been discussed. Eddy [127] focused on the inability of *A. salmonicida *to produce 2,3-butanediol from glucose, and the absence of motility, which were in contrast to the genus description [128]. A new genus, i.e. *Necromonas*, was proposed with two species, namely *N. salmonicida *for the typical isolates and *N. achromogenes *for the non-pigmented strains [118]. This proposal was not formally widely accepted, although Cowan [129] used the suggestion in his landmark identification scheme for medically important bacteria. Subsequent serological and bacteriophage sensitivity data supported the relationship between *A. salmonicida *and the motile aeromonads. Common antigens between *A. hydrophila *and *A. salmonicida *subsp. *masoucida *and other isolates of *A. salmonicida *were reported [122,130]. Furthermore, serological cross-reactions between *A. salmonicida *and motile aeromonads were discussed [131]. Moreover, *A. hydrophila *cultures were found to be sensitive to *A. salmonicida *bacteriophages [132,133]. The outcome of all the studies is that DNA homology supports the classification of *A. salmonicida *in the genus *Aeromonas *(e.g. [92,122,126]).

There are certainly outstanding questions about the validity and taxonomic placing of *Haemophilus piscium *[123], but an authentic reference strain was not deposited any in any recognized culture collection at the time of its first isolation. Later, it was concluded that the organism was not a *bona fide Haemophilus *because of the lack of requirement for haemin or NAD [134]. In particular *H. piscium *differed from the type species of the genus, *H. influenzae*, in the inability to reduce nitrate or alkaline phosphatase and to grow at 37°C, in conjunction with a higher G+C ratio of the DNA. It was commented that there was only a low similarity between *H. piscium *and other *Haemophilus *spp. in a numerical taxonomic study [135]. A link with atypical, achromogenic *A. salmonicida *was made [122]. This link was reinforced by bacteriophage sensitivity, when it was concluded that *H. piscium *is an atypical *A. salmonicida *[136]. Other workers have supported this view (e.g. [114]). However with the absence of an authentic, original type strain, the definitive taxonomic position of *H. piscium *is only speculative.

A lack of congruence has been reported between the results of molecular (PCR, RAPD and ribotyping) and phenotypic methods in taxonomy of aeromonads [114]. Moreover, there are problems of inter-laboratory differences and lack of standardisation in test methods [137]. The outcome is that the definitive classification of *A. salmonicida *has not been achieved, to date.

### Enteric redmouth (ERM)

There has been discussion about the taxonomic position of the aetiological agent of ERM. Strong agglutination with *Salmonella enterica *subsp *arizonae *O group 26, and a weak reaction with O group 29 was reported [138]. In addition, biochemical similarities with enterics, notably *Enterobacter liquefaciens, Serratia marcescens *subsp. *kiliensis *as well as *Salmonella enterica *subsp. *arizonae *were mentioned [138]. Serological cross-reactions were also recorded with *Hafnia alvei *[139]. Nevertheless, a new species, i.e. *Yersinia ruckeri *was described although there was only a 30-31% DNA homology with *Y. enterocolitica *and *Y. pseudotuberculosis *[140]. This compares to DNA homologies of 24-28% and 31% with *Serratia marcescens *and *Serratia liquefaciens*, respectively [141]. Indeed, it has been suggested that the causal agent of ERM should belong in a new genus of the Enterobacteriaceae [142]. A complication developed when a new non-motile form of the pathogen was recovered from rainbow trout. By 16S rRNA sequencing and a homology of 100%, the organisms were linked to *Y. ruckeri *but regarded as a new biogroup [143]. Similar non-motile variants were also recovered from previously vaccinated rainbow trout in Spain [144].

### Vibriosis

The causal agent of "red-pest" in eels was first designated as *Bacterium anguillarum *[6]. Subsequently, an outbreak among eels in Sweden led to the use of the name *Vibrio anguillarum*. Numerous studies have pointed to heterogeneity in *V. anguillarum *initially with the delineation of two sub-groupings/biotypes (e.g. [12]). This increased to 3 [145] and then 4 sub-groups/phena within the species definition [146,147]. Ribotyping has confirmed the heterogeneity [148], although a single taxon, homogeneous by ribotyping but heterogeneous by LPS profiles, plasmid composition, serogrouping, and BIOLOG-GN fingerprints and API 20E profiles was described [149,150]. Biotype II became recognized as a separate species, i.e. *V. ordalii *[151], which is homogeneous by plasmid profiling, ribotyping and serogrouping, accommodates two LPS groups, but is heterogeneous by BIOLOG-GN fingerprints and API 20E profiles [150].

Serology has been widely used for diagnosis, but has complicated the understanding of *V. anguillarum *[151], and the establishment of serotypes has to some extent traversed species boundaries. With *V. anguillarum*, serogroup 02 and 05, there are common antigens with *V. ordalii *[152] and *V. harveyi *[149], respectively. Initially, three serotypes were recognised for isolates from salmonids from the northwest USA, Europe, and the Pacific-northwest (USA) [103]. This number increased to 6 [128], and then 10 [153] and upwards [148,154]. Serogroup O1 dominates the number of isolates available for study and the relative importance to fish pathology [149,155-157]. Serogroup O2 has been further subdivided into serogroup O2a and O2b [158].

*V. anguillarum *was re-classified initially to *Beneckea *[12] and then to a newly proposed genus *Listonella *[13], but the changes were not widely accepted.

## The role of phylogenetics in bacterial fish pathology

The techniques described above are relevant for the taxonomy of bacterial fish pathogens. Yet, molecular methods, namely sequencing of the 16S rRNA gene, permit the study of evolutionary relationships, i.e. phylogenetics, which may be viewed as phylogenetic trees, which are interpreted by cladistics and used in defining taxa. The approach is essential in the study of the evolutionary tree of life, but is it strictly necessary for fish pathology and the recognition of species? One concern is the comparative fluidity by which genes may be exchanged, such as by horizontal gene transfer, and the impact of this movement on the outcome of the taxonomic/phylogenetic process.

## Conclusions

There has been a resurgence of interest in bacterial taxonomy partially because of the current focus on biodiversity and the development of reliable molecular methods [159], notably 16S rRNA sequencing. Undoubtedly, these molecular approaches have led to greater confidence and accuracy in the reporting of bacterial names. Nevertheless, it is conceded that bacterial taxonomy is a specialist subject, which is not of interest to all fish pathologists. However, it cannot be overstated that there is a real value for good taxonomy as a means of communication. In terms of fish pathology, taxonomy enables the recognition of new pathogens, improvements in the understanding of relationships between taxa, an appreciation of variation within existing nomenspecies including the recognition of new subspecies and biogroups, and facilitates accurate commentary about all aspects of biology from epizootiology to pathogenicity (e.g. [160]), although the position of so-called atypical isolates in taxonomic hierarchies is often difficult to determine [161].

For the future, a range of new techniques, including in situ hybridization, probe hybridization, microarray techniques and restriction enzyme digestion, are entering taxonomic use, and are likely to be used in fish pathology. The impact of these new approaches is difficult to predict, but will undoubtedly be incorporated in some fish bacteriology laboratories.

## Competing interests

The author declares that they have no competing interests.

## References

[B1] Sneath PHA The application of computers to taxonomy J Gen Microbiol 1957 17 201 226 13475686 10.1099/00221287-17-1-201

[B2] Sokal RR Michener CD Numerical Taxonomy 1958 WH Freeman, London

[B3] Arakawa CK Fryer JL Isolation and characterization of a new subspecies of *Mycobacterium chelonei *infections for salmonid fish Helgoländer Meeresuntersuch 1984 37 329 342

[B4] Brennan PJ Ratledge C, Wilkinson SG *Mycobacterium *and other actinomycetes Microbial Lipids 1988 1 Academic Press, London 203 298

[B5] Kämpfer P The characterization of prokaryote strains for taxonomic purposes Int J Syst Evol Microbiol 2010 60 7 20068236 10.1099/ijs.0.020768-0

[B6] Austin B Austin DA Bacterial Fish Pathogens: Disease in Farmed and Wild Fish 2007 4 Springer-Praxis, Goldalming

[B7] Schiewe MH Trust TJ Crosa JH *Vibrio ordalii *sp. nov.: a causative agent of vibriosis in fish Curr Microbiol 1981 6 343 348 10.1007/BF01567009

[B8] Schmidtke LM Carson J Characteristics of *Vagococcus salmoninarum *isolated from diseased salmonid fish J Appl Bacteriol 1994 77 229 236 7961194 10.1111/j.1365-2672.1994.tb03068.x

[B9] Gauger E Gómez-Chiarri M 16S ribosomal DNA sequencing confirms the synonymy of *Vibrio harveyi *and *V. carchariae* Dis Aquat Org 2002 52 39 46 10.3354/dao052039 12517004

[B10] Pedersen K Verdonck L Austin B Austin DA Blanch AR Grimont PAD Jofre J Koblavi S Larsen JL Tiainen T Vigneulle M Swings J Taxonomic evidence that *Vibrio carchariae *Grimes et al. 1985 is a junior synonym of *Vibrio harveyi *(Johnson and Shunk 1936) Baumann et al. 1981 Int J Syst Bacteriol 1998 48 749 758 10.1099/00207713-48-3-749

[B11] Thompson FL Hoste B Vandemeulebroecke K Engelbeen K Denys R Swings J *Vibrio trachuri *Iwamoto et al., 1995 is a junior synonym of *Vibrio harveyi *(Johnson and Shunk 1936) Baumann et al. 1981 Int J Syst Evol Microbiol 2002 52 973 976 10.1099/ijs.0.02046-0 12054265

[B12] Baumann P Bang SS Baumann L Phenotypic characterization of *Beneckea anguillara *biotypes I and II Curr Microbiol 1978 1 85 88 10.1007/BF02605421

[B13] MacDonell MT Colwell RR Phylogeny of the Vibrionaceae and recommendations for two new genera, *Listonella *and *Shewanella* Syst Appl Microbiol 1985 6 171 182

[B14] Vandamme P Pot B Gillis M de Vos P Kersters K Swings J Polyphasic taxonomy, a consensus approach to bacterial systematics Microbiol Rev 1996 60 407 438 8801440 10.1128/mr.60.2.407-438.1996 PMC239450

[B15] Toranzo AE Santos Y Nieto TP Barja JL Evaluation of different assay systems for identification of environmental *Aeromonas *strains Appl Environ Microbiol 1986 51 652 656 16347025 10.1128/aem.51.3.652-656.1986 PMC238934

[B16] Sneath PHA Johnson R The influence of numerical taxonomic similarities of errors in microbiological tests J Gen Microbiol 1972 72 377 392 4562310 10.1099/00221287-72-2-377

[B17] Austin B Embley TM Goodfellow M Selective isolation of *Renibacterium salmoninarum* FEMS Microbiol Lett 1983 17 111 114 10.1111/j.1574-6968.1983.tb00383.x

[B18] Walters GR Plumb JA Modified oxidation/fermentation medium for use in the identification of bacterial fish pathogens J Fish Res Bd Can 1978 35 1629 1630

[B19] Adams A Thompson KD Recent applications of biotechnology to novel diagnostics for aquatic animals Rev Sci Tech 2008 27 197 209 18666488

[B20] Goerlich R Schlüsener HJ Lehmann J Greuel E The application of monoclonal antibodies to diagnosis of *Aeromonas salmonicida *infections in fishes Bull Europ Assoc Fish Pathol 1984 4 66

[B21] Jung TS Thompson KD Adams A Morris DJ Snedden K The production of monoclonal antibodies against *Photobacterium damselae *subsp. *piscicida *and their application in immunohistochemistry J Fish Dis 2001 24 64 67 10.1046/j.1365-2761.2001.00260.x

[B22] Steiropoulos NA Yuksel SA Thompson KD Adams A Ferguson HW Detection of *Rickettsia*-like organisms (RLOs) in European sea bass *(Dicentrarchus labrax*, L) by immunohistochemistry, using rabbit anti-*Rickettsia salmonis *serum Bull Eur Assoc Fish Pathol 2002 22 428 432

[B23] Adams A Thompson KD Morris D Farias C Chen SC Development and use of monoclonal antibody probes for immunohistochemistry, ELISA and IFAT to detect bacterial and parasitic fish pathogens Fish Shellfish Immunol 1995 5 537 547 10.1016/S1050-4648(95)80040-9

[B24] Klesius P Evans J Shoemaker C Yeh H Goodwin AE Adams A Thompson KD Rapid detection and identification of *Streptococcus iniae *using a monoclonal antibody-based indirect fluorescent antibody technique Aquaculture 2005 258 180 186 10.1016/j.aquaculture.2005.06.040

[B25] Adams A Burnell G, Allan G Advances in disease diagnosis, vaccine development and other emerging methods to control pathogens 2009 Chapter 7 New Technologies in Aquaculture, Improving Production, Efficiency, Quality and Environmental Management, Woodhead, England 197 211

[B26] Saeed MO Plumb JA Serological detection of *Edwardsiella ictaluri *Hawke lipopolysaccharide antibody in serum of channel catfish *Ictalurus punctatus *Rafinesque J Fish Dis 1987 10 205 209 10.1111/j.1365-2761.1987.tb01062.x

[B27] Adkinson MA Gilad O Hedricks RP An enzyme linked immunosorbent assay (ELISA) for detection of antibodies to the Koi herpes virus (KHV) in the serum of Koi *Cyprinus carpio* Fish Pathol 2005 40 53 62

[B28] Mayer H Masoud H Urbanik-Sypniewska T Weckesser J Lipid A composition and phylogeny of Gram-negative bacteria Bull Jap Fed Cult Collect 1989 5 19 25

[B29] Scheifer KH Classification of bacteria and Archaea: past, present and future Syst Appl Microbiol 2009 32 533 542 10.1016/j.syapm.2009.09.002 19819658

[B30] Rhodes MW Kator H Kotob S van Berkum P Kaattari I Vogelbein W Quinn F Floyd MM Butler WR Ottinger CA *Mycobacterium shottsii *sp nov., a slowly growing species isolated from Chesapeake Bay striped bass (*Morone saxatilis)* Int J Syst Evol Microbiol 2003 53 421 424 10.1099/ijs.0.02299-0 12710607

[B31] Tindall BJ Rosselló-Móra R Busse HJ Ludwig W Kämpfer P Notes on the characterization of prokaryote strains for taxonomic purposes Int J Syst Evol Microbiol 2010 60 249 266 10.1099/ijs.0.016949-0 19700448

[B32] Williams AM Fryer JL Collins MD *Lactococcus piscium *sp. nov. a new *Lactococcus *species from salmonid fish FEMS Microbiol Lett 1990 68 109 114 10.1111/j.1574-6968.1990.tb04132.x 1692000

[B33] Backman S Ferguson HW Prescott JF Wilcock BP Progressive panophthalmitis in chinook salmon, *Oncorhynchus tshawytscha *(Walbaum): a case report J Fish Dis 1990 13 345 353 10.1111/j.1365-2761.1990.tb00793.x

[B34] Isik K Chun J Hah YC Goodfellow M *Nocardia salmonicida *nom. rev., a fish pathogen Int J Syst Bacteriol 1999 49 833 837 10.1099/00207713-49-2-833 10319509

[B35] Fiedler F Draxl R Biochemical and immunochemical properties of the cell surface of *Renibacterium salmoninarum* J Bacteriol 1986 168 799 804 3782026 10.1128/jb.168.2.799-804.1986 PMC213555

[B36] Sanders JE Fryer JL *Renibacterium salmoninarum *gen. nov., sp. nov., the causative agent of bacterial kidney disease in salmonid fishes Int J Syst Bacteriol 1980 30 496 502 10.1099/00207713-30-2-496

[B37] Embley TM Goodfellow M Minnikin DE Austin B Fatty acid, isoprenoid quinone and polar lipid composition in the classification of *Renibacterium salmoninarum* J Appl Bacteriol 1983 55 31 37

[B38] Fox GE Pechman KR Woese CR Comparative cataloging of 16S ribosomal ribonucleic acid: molecular approach to procaryotic systematics Int J Syst Bacteriol 1977 27 44 57 10.1099/00207713-27-1-44

[B39] Lane DJ Field KG Olsen GJ Pace NR Reverse transcriptase sequencing of ribosomal RNA for phylogenetic analysis Meth Enzymol 1988 167 138 144 full_text 2467178 10.1016/0076-6879(88)67015-7

[B40] Sanger F Nicklen S Coulson AR DNA sequencing with chain-terminating inhibitors Proc Natl Acad Sci USA 1977 74 5463 5467 10.1073/pnas.74.12.5463 271968 PMC431765

[B41] Saiki RK Gelfand DH Stoffel S Scharf SJ Higuchi R Horn GT Mullis KB Erlich HA Primer-directed enzymatic amplification of DNA with a thermostable DNA polymerase Science 1988 239 487 491 10.1126/science.2448875 2448875

[B42] De Ley J Park IW Tijtgat R van Ermengem J DNA homology and taxonomy of *Pseudomonas *and *Xanthomonas* J Gen Microbiol 1966 42 43 56 5922298 10.1099/00221287-42-1-43

[B43] Fox GE Wisotzkey JD Jurtshuk P Jr How close is close: 16S rRNA sequence identity may not be sufficient to guarantee species identity Int J Syst Bacteriol 1992 42 166 170 10.1099/00207713-42-1-166 1371061

[B44] Kvitt H Colorni A Strain variation and geographic endemism in *Streptococcus iniae* Dis Aquat Org 2004 61 67 73 10.3354/dao061067 15584412

[B45] Gauthier G Lafay B Ruimy R Breittmayer V Nicolas JL Gauthier M Christen R Small-subunit rRNA sequences and whole DNA relatedness concur for the re-assignment of *Pasteurella piscicida *(Snieszko et al.) Janssen and Surgalla to the genus *Photobacterium *as *Photobacterium damsela *subsp. *piscicida *comb. nov Int J Syst Bacteriol 1995 45 139 144 10.1099/00207713-45-1-139 7531996

[B46] Gutenberger SK Giovannoni SJ Field KG Fryer JL Rohovec JS A phylogenetic comparison of the 16S rRNA sequence of the fish pathogen, *Renibacterium salmoninarum*, to Gram-positive bacteria FEMS Microbiol Lett 1991 77 151 156 10.1111/j.1574-6968.1991.tb04339.x 1709893

[B47] Stackebrandt E Wehmeyer U Nader H Fiedler F Phylogenetic relationship of the fish pathogenic *Renibacterium salmoninarum *to *Arthrobacter, Micrococcus *and related taxa FEMS Microbiol Lett 1988 50 117 120 10.1111/j.1574-6968.1988.tb02922.x

[B48] Wiens GD Rockey DD Wu ZN Chang J Levy R Crane S Chen DS Capri GR Burnett JR Sudheesh PS Schipma MJ Burd H Bhattacharyya A Rhodes LD Kaul R Strom MS Genome sequence of the fish pathogen *Renibacterium salmoninarum *suggests reductive evolution away from an environmental *Arthrobacter *ancestor J Bacteriol 2008 190 6970 6982 10.1128/JB.00721-08 18723615 PMC2580684

[B49] Ransangan J Mustafa S Identification of *Vibrio harveyi *isolated from diseased Asian sea bass *Lates calcarifer *by use of 16S ribosomal DNA sequencing J Aquat Anim Health 2009 21 150 155 10.1577/H09-002.1 20043399

[B50] Nomoto R Munasinghe LI Shimahara Y Yasuda H Nakamura A Misawa N Itami T Yoshida T Lancefield group C *Streptococcus dysgalactiae *infection responsible for fish mortalities in Japan J Fish Dis 2004 27 679 686 10.1111/j.1365-2761.2004.00591.x 15575875

[B51] Doménech A Fernández-Garayzábal JF Pascual C Garcia JA Cutuli MT Moreno MA Collins MD Dominguez L Streptococcosis in cultured turbot, *Scophthalmus maximus *(L.), associated with *Streptococcus parauberis* J Fish Dis 1996 19 33 38

[B52] Wallbanks S Martinez-Murcia AJ Fryer JL Phillips BA Collins MD 16S rRNA sequence determination for members of the genus *Carnobacterium *and related lactic acid bacteria and description of *Vagococcus salmoninarum *sp. nov Int J Syst Bacteriol 1990 40 224 230 10.1099/00207713-40-3-224 1697764

[B53] Chen SC Liaw LL Su HY Ko SC Wu CY Chaung HC Tsai YH Yang KL Chen YC Chen TH Lin GR Cheng SY Lin YD Lee JL Lai CC Weng YJ Chu SY *Lactococcus garvieae*, a cause of disease in grey mullet, *Mugil cephalus *L., in Taiwan J Fish Dis 2002 25 727 732 10.1046/j.1365-2761.2002.00415.x

[B54] Hiney MP Smith PR Validation of polymerase chain reaction-based techniques for proxy detection of bacterial fish pathogens: framework, problems and possible solutions for environmental applications Aquaculture 1998 162 41 68 10.1016/S0044-8486(98)00207-5

[B55] Knibb W Colorni A Ankaoua M Lindell D Diamant A Gordin H Detection and identification of a pathogenic marine mycobacterium from the European seabass *Dicentrarchus labrax *using polymerase chain reaction and direct sequencing of 16S rRNA sequences Mol Mar Biol Biotechnol 1993 2 225 232 8293073

[B56] McCormick JI Hughes MS McLoughlin MF Identification of *Mycobacterium chelonae *in a cichlid oscar *Astronotus ocellatus *Cuvier, by direct cycle sequencing of polymerase chain reaction amplified 16S rRNA gene sequences J Fish Dis 1995 18 459 461 10.1111/j.1365-2761.1995.tb00337.x

[B57] Zlotkin A Eldar A Ghittino C Bercovier H Identification of *Lactococcus garvieae *by PCR J Clin Microbiol 1998 36 983 985 9542921 10.1128/jcm.36.4.983-985.1998 PMC104673

[B58] Mata AI Gibello A Casamayor A Blanco MM Domínquez L Fernández-Garayzábal JF Multiplex PCR assay for the detection of bacterial pathogens associated with warm-water streptococcosis in fish Appl Environ Microbiol 2004 70 3183 3187 10.1128/AEM.70.5.3183-3187.2004 15128589 PMC404434

[B59] Aoki T Park CI Yamashita H Hirono I Species-specific polymerase chain reaction primers for *Lactococcus garvieae* J Fish Dis 2000 23 1 6 10.1046/j.1365-2761.2000.00207.x

[B60] León G Maulén N Figueroa J Villanueva J Rodríguez C Vera MI Krauskopf M A PCR based assay for the identification of the fish pathogen *Renibacterium salmoninarum* FEMS Microbiol Lett 1994 115 131 136 8138127 10.1111/j.1574-6968.1994.tb06627.x

[B61] León G Martinez MA Etchegaray JP Vera MI Figueroa J Krauskopf M Specific DNA probes for the identification of the fish pathogen, *Renibacterium salmoninarum* Wld J Microbiol Biotechnol 1994 10 149 153 10.1007/BF00360876 24420936

[B62] Magnüssen HB Fridjónsson ÓH Andrésson ÓS Benediktsdóttir E Gudmundsdóttir S Andrésdottír V *Renibacterium salmoninarum*, the causal agent of bacterial kidney disease in salmonid fish, detected by nested reverse transcription-PCR of 16S rRNA sequences Appl Environ Microbiol 1994 60 4580 4583 7529017 10.1128/aem.60.12.4580-4583.1994 PMC202022

[B63] Miyoshi Y Suzuki S PCR method to detect *Nocardia seriolae *in fish samples Fish Pathol 2003 38 93 97

[B64] Soria-Carrasco V Talavera G Igea J Castresana J The K tree score: quantification of differences in the relative branch length and topology of phylogenetic trees Bioinformatics 2007 23 2954 2956 10.1093/bioinformatics/btm466 17890735

[B65] Maher M Palmer R Gannon F Smith T Relationship of a novel bacterial fish pathogen to *Streptobacillus moniliformis *and the fusobacteria group, based on 16S ribosomal RNA analysis Syst Appl Microbiol 1995 18 79 84

[B66] Sakai M Konno T Tassakka ACMAR Ponpornpisit A Areechon N Katagiri T Yoshida T Endo M Characterization of a *Mycobacterium *sp. isolated from guppy *Poecilia reticulata*, using 16S ribosomal RNA and its internal transcribed spacer sequences Bull Eur Assoc Fish Pathol 2005 25 64 69

[B67] Pourahmad F Cervellione F Thompson KD Taggart JB Adams A Richards RH *Mycobacterium stomatepiae *sp. nov., a slowly growing non-chromogenic species isolated from fish Int J Syst Evol Microbiol 2008 58 2821 2827 10.1099/ijs.0.2008/001164-0 19060066

[B68] Martinez-Murcia AJ Esteve C Garay E Collins MD *Aeromonas allosaccharophila *sp. nov., a new mesophilic member of the genus *Aeromonas* FEMS Microbiol Lett 1992 91 199 206 10.1111/j.1574-6968.1992.tb05209.x 1378035

[B69] Huys G Kämpfer P Swings J New DNA-DNA hybridization and phenotypic data on the species *Aeromonas ichthiosmia *and *Aeromonas allosaccharophila*: A. *ichthiosmia is a later synonym of A. veronii *Hickman-Brenner et al. 1987 Syst Appl Microbiol 2001 24 177 182 10.1078/0723-2020-00038 11518320

[B70] Mikalsen J Olsen AB Tengs T Colquohoun DJ *Francisella philomiragia *subsp *noatunensis *subsp nov., isolated from farmed Atlantic cod (*Gadus morhua *L.) Int J Syst Evol Microbiol 2007 57 1960 1965 10.1099/ijs.0.64765-0 17766855

[B71] Nylund A Ottem KF Watanabe K Karlsbakk E Krossoy B *Francisella *sp (Family Francisellaceae) causing mortality in Norwegian cod *(Gadus morhua) *farming Arch Microbiol 2006 185 383 392 10.1007/s00203-006-0109-5 16614828

[B72] Birkbeck TH Laidler LA Grant AN Cox DI *Pasteurella skyensis *sp nov., isolated from Atlantic salmon *(Salmo salar *L.) Int J Syst Evol Microbiol 2002 52 699 704 10.1099/ijs.0.01884-0 12054228

[B73] Fryer JL Lannan CN Giovannoni SJ Wood ND *Piscirickettsia salmonis *gen. nov., the causative agent of an epizootic disease in salmonid fishes Int J Syst Bacteriol 1992 42 120 126 10.1099/00207713-42-1-120 1371057

[B74] Arkush KD Mcbride AM Mendonca HL Okihiro MS Andree KB Marshall S Henriquez V Hedrick RP Genetic characterization and experimental pathogenesis of *Piscirickettsia salmonis *isolated from white seabass *Atractascion nobilis* Dis Aquat Org 2005 63 139 149 10.3354/dao063139 15819429

[B75] Corbeil S Hyatt AD Crane MStJ Characterisation of an emerging rickettsia-like organism in Tasmanian farmed Atlantic salmon *Salmo salar* Dis Aquat Org 2005 64 37 44 10.3354/dao064037 15900686

[B76] Nishimori E Kita-Tsukamoto K Wakabayashi H *Pseudomonas plecoglossicida *sp nov., the causative agent of bacterial haemorrhagic ascites of ayu, *Plecoglossus altivelis* Int J Syst Evol Microbiol 2000 50 83 89 10826790 10.1099/00207713-50-1-83

[B77] Romalde JL Ravelo C Valdes I Magariños B de la Fuente E Martin CS Avendano-Herrera R Toranzo AE *Streptococcus phocae*, an emerging pathogen for salmonid culture Vet Microbiol 2008 130 198 207 10.1016/j.vetmic.2007.12.021 18280675

[B78] Pineiro-Vidal M Carballas C Gomez-Barreiro O Riaza A Santos Y *Tenacibaculum soleae *sp nov., isolated from diseased sole *(Soleae senegalensis *Kaup) Int J Syst Evol Microbiol 2008 58 881 885 10.1099/ijs.0.65539-0 18398187

[B79] Lunder T Sorum H Holstad G Steigerwalt AG Mowinckel P Brenner DJ Phenotypic and genotypic characterization of *Vibrio viscosus *sp nov and *Vibrio wodanis *sp nov isolated from Atlantic salmon *(Salmo salar) *with "winter ulcer" Int J Syst Evol Microbiol 2000 50 427 450 10758846 10.1099/00207713-50-2-427

[B80] Benediktsdóttir E Verdonck L Spröer C Helgason S Swings J Characterization of *Vibrio viscosus *and *Vibrio wodanis *isolated from different geographical locations: a proposal for re-classification of *Vibrio viscosus *as *Moritella viscose *comb. nov Int J Syst Evol Microbiol 2000 50 479 488 10758850 10.1099/00207713-50-2-479

[B81] Benediktsdóttir E Helgason S Sigurjónsdóttir H *Vibrio *spp. isolated from salmonids with shallow skin lesions and reared at low temperature J Fish Dis 1988 21 19 28 10.1046/j.1365-2761.1998.00065.x 29739170

[B82] Urakawa H Kita-Tsukamoto K Stevens SE Ohwada K Colwell RR A proposal to transfer *Vibrio marinus *(Russell 1891) to a new genus *Moritella *gen. nov. as *Moritella marina *comb. nov FEMS Microbiol Lett 1998 165 373 378 10.1111/j.1574-6968.1998.tb13173.x 9742712

[B83] Del-Pozo J Crumlish M Ferguson HW Turnbull JF Retrospective cross-sectional study on "*Candidatus arthromitus*" associated rainbow trout gastroenteritis (RTGE) in the UK Aquaculture 2009 290 22 27 10.1016/j.aquaculture.2009.02.009

[B84] Michel C Bernardet JF Daniel P Chilmonczyk S Urdaci M de Kinkelin P Clinical and aetiological aspects of a summer enteritic syndrome associated with the sporulating segmented filamentous bacterium "*Candidatus Arthromitus*" in farmed rainbow trout, *Oncorhynchus mykiss *(Walbaum) J Fish Dis 2002 25 533 543 10.1046/j.1365-2761.2002.00400.x

[B85] Del-Pozo J Turnbull J Ferguson H Crumlish M A comparative molecular study of the presence of "*Candidatus arthromitus*" in the digestive system of rainbow trout, *Oncorhynchus mykiss *(Walbaum), healthy and affected with rainbow trout gastroenteritis J Fish Dis 2010 33 241 250 10.1111/j.1365-2761.2009.01117.x 19912454

[B86] Stevenson RMW Daly JG Biochemical and serological characteristics of Ontario isolates of *Yersinia ruckeri* Can J Fish Aquat Sci 1982 39 870 876

[B87] Karlsen M Nylund A Watanabe K Helvik JV Nylund S Plarre H Characterization of "*Candidatus Clavochlamydia salmonicola*": an intracellular bacterium infecting salmonid fish Environ Microbiol 2008 10 208 218 17894816 10.1111/j.1462-2920.2007.01445.x

[B88] Larsen JL Jensen NJ An *Aeromonas *species implicated in ulcer-disease of the cod (*Gadus morhua*) Nord Veterinaermed 1977 29 199 211 325501

[B89] Orozova P Barker M Austin DA Austin B Identification and pathogenicity to rainbow trout, *Oncorhynchus mykiss *(Walbaum), of some aeromonads J Fish Dis 2009 32 865 871 10.1111/j.1365-2761.2009.01065.x 19602093

[B90] Carnahan AM Altwegg M Austin B, Altwegg M, Gosling PJ, Joseph S, Wiley, Chichester Taxonomy The Genus Aeromonas 1996 1 38

[B91] Allen DA Austin B Colwell RR *Aeromonas media*, a new species isolated from river water Int J Syst Bacteriol 1983 33 599 604 10.1099/00207713-33-3-599

[B92] MacInnes JI Trust TJ Crosa JH Deoxyribonucleic acid relationships among members of the genus *Aeromonas* Can J Microbiol 1979 25 579 586 10.1139/m79-083 476540

[B93] Esteve C Numerical taxonomy of *Aeromonadaceae *and *Vibrionaceae *associated with reared fish and surrounding fresh and brackish water System Appl Microbiol 1995 18 391 402

[B94] Esteve C Biosca EG Amaro C Virulence of *Aeromonas hydrophila *and some other bacteria isolated from European eels *Anguilla anguilla *reared in fresh water Dis Aquat Org 1993 16 15 20 10.3354/dao016015

[B95] Ali A Carnahan AM Altwegg M Lüthy-Hotenstein J Joseph SW *Aeromonas bestiarum *sp. nov. (formerly genomospecies DNA group 2 *A. hydrophila) *a new species isolated from non-human sources Med Microbiol Lett 1996 5 156 165

[B96] Toranzo AE Baya AM Romalde JJ Hetrick FM Association of *Aeromonas sobria *with mortalities of adult gizzard shad, *Dorosoma cepedianum *Lesueur J Fish Dis 1989 12 439 448 10.1111/j.1365-2761.1989.tb00555.x

[B97] Ma Z Yang H Li T Luo L Gao JL Isolation and identification of pathogenic *Aeromonas veronii *isolated from infected Siberian sturgeon (*Acipenser baerii*) Wei Sheng Wu Xue Bao 2009 49 1289 1294 (article in Chinese) 20069873

[B98] Snieszko SF Breed RS, Murray EGD, Smith NR Genus IV. *Aeromonas *Kluyver and van Niel 1936, Bergey's Manual of Determinative Bacteriology 1957 7 Williams and Wilkins, Baltimore 189 193

[B99] Colwell RR MacDonell MT De Ley J Proposal to recognize the family Aeromonadaceae fam. nov Int J Syst Bacteriol 1986 36 473 477 10.1099/00207713-36-3-473

[B100] Griffin PJ Snieszko SF Friddle SB A more comprehensive description of *Bacterium salmonicida* Trans Am Fish Soc 1953 82 129 138 10.1577/1548-8659(1952)82[129:AMCDOB]2.0.CO;2

[B101] Austin DA McIntosh D Austin B Taxonomy of fish associated *Aeromonas *spp., with the description of *Aeromonas salmonicida *subsp. *smithia *subsp. nov Syst Appl Microbiol 1989 11 277 290

[B102] Duff DCB Dissociation in *Bacillus salmonicida*, with special reference to the appearance of the G form of culture J Bacteriol 1937 34 49 67 16560037 10.1128/jb.34.1.49-67.1937 PMC545187

[B103] Pacha RE Kiehn ED Characterization and relatedness of marine vibrios pathogenic to fish: physiology, serology and epidemiology J Bacteriol 1969 100 1242 1247 5391230 10.1128/jb.100.3.1242-1247.1969 PMC250304

[B104] Koppang EO Fjølstad M Melgård B Vigerust M Sørum H non-pigment-producing isolates of *Aeromonas salmonicida *subspecies *salmonicida: *isolation, identification, transmission and pathogenicity in Atlantic salmon, *Salmo salar *L J Fish Dis 2000 23 39 48 10.1046/j.1365-2761.2000.00205.x

[B105] Nakatsugawa T Atypical *Aeromonas salmonicida *isolted from cultured shotted halibut Fish Pathol 1994 29 193 198

[B106] Kaku Y Yamada Y Wakabayashi H Characterization of atypical *Aeromonas salmonicida *isolated from an epizootic ulcerative disease in carp (*Cyprinus carpio*) Fish Pathol 1999 34 155 162

[B107] Wiklund T Bylund G Skin ulcer disease of flounder *Platichthys flesus *in the northern Baltic Sea Dis Aquat Org 1993 17 165 174 10.3354/dao017165

[B108] Austin B Recovery of "atypical" isolates of *Aeromonas salmonicida*, which grow at 37°C, from ulcerated non-salmonids in England J Fish Dis 1993 16 165 168 10.1111/j.1365-2761.1993.tb00862.x

[B109] Goodwin AE Merry GE Are all Koi ulcer cases associated with infection by atypical *Aeromonas salmonicida*? Polymerase chain reaction assays of Koi carp skin swabs submitted by hobbyists J Aquat Anim Health 2009 21 98 103 10.1577/H08-042.1 19873831

[B110] Pedersen K Kofod H Dalsgaard I Larsen JL Isolation of oxidase-negative *Aeromonas salmonicida *from disease turbot *Scophthalmus maximus* Dis Aquat Org 1994 18 149 154 10.3354/dao018149

[B111] Wiklund T Dalsgaard I Eerola E Olivier G Characteristics of "atypical" cytochrome-oxidase negative *Aeromonas salmonicida *isolated from ulcerated flounder *(Platichthys flesus *(L.) J Appl Bacteriol 1994 76 511 520 8005836 10.1111/j.1365-2672.1994.tb01110.x

[B112] Wiklund T Dalsgaard I Atypical *Aeromonas salmonicida *associated with ulcerated flatfish species in the Baltic Sea and the North Sea J Aquat Anim Health 1995 7 218 224 10.1577/1548-8667(1995)007<0218:AASAWU>2.3.CO;2

[B113] Goldschmidt-Clermont E Hochwartner O Demarta A Caminada AP Frey J Outbreaks of an ulcerative and haemorrhagic disease in Arctic char *Salvelinus alpinus *caused by *Aeromonas salmonicida *subsp *smithia* Dis Aquat Org 2009 86 81 86 10.3354/dao02110 19899353

[B114] Austin B Austin DA Dalsgaard I Gudmundsdóttir BK Høie S Thornton JM Larsen JL O'Hici B Powell R Characterization of atypical *Aeromonas salmonicida *by different methods Syst Appl Microbiol 1998 21 50 64 9741110 10.1016/s0723-2020(98)80008-8

[B115] Høie S Dalsgaard I Aase IL Heum M Thornton JM Powell R Polymerase chain reaction (PCR) based typing analysis of atypical isolates of the fish pathogen *Aeromonas salmonicida* Syst Appl Microbiol 1999 22 403 411 10553293 10.1016/S0723-2020(99)80049-6

[B116] Lund V Jenssen LM Wesmajervi MS Assessment of genetic variability and relatedness among atypical *Aeromonas salmonicida *from marine fishes, using AFLP fingerprinting Dis Aquat Org 2002 50 119 126 10.3354/dao050119 12180702

[B117] Yamada Y Kaku Y Wakabayashi H Phylogenetic intrarelationships of atypical *Aeromonas salmonicida *isolated in Japan as determined by 16S rDNA sequencing Fish Pathol 2000 35 35 40

[B118] Smith IW The classification of *Bacterium salmonicida* J Gen Microbiol 1963 33 263 274 14121202 10.1099/00221287-33-2-263

[B119] Kimura T A new subspecies of *Aeromonas salmonicida *as an etiological agent of furunculosis on "Sakuramasu" *(Oncorhynchus masou) *and pink salmon *(O. gorbuscha) *rearing for maturity Fish Pathol 1969 3 34 44 Part 1. On the serological properties

[B120] Schubert RHW Buchanan RE, Gibbons NE Genus II. *Aeromonas *Kluyver and van Niel 1936, 398, Bergey's Manual of Determinative Bacteriology 1974 8th Williams and Wilkins, Baltimore 345 348

[B121] Popoff M Bacterioses, generalities - *Aeromonas *et Aeromonose. Mimeograph 1970 Institut National de la Recherche Agronomique 1 38

[B122] Paterson WD Douey D Desautels D Relationships between selected strains of typical and atypical *Aeromonas salmonicida, Aeromonas hydrophila*, and *Haemophilus piscium* Can J Microbiol 1980 26 588 598 10.1139/m80-104 7397604

[B123] Snieszko SF Griffin P Friddle SB A new bacterium *(Haemophilus piscium *n. sp.) from ulcer disease of trout J Bacteriol 1950 59 699 710 15436444 10.1128/jb.59.6.699-710.1950 PMC385819

[B124] Thornton JM Austin DA Austin B Powell R Small subunit rRNA gene sequences of *Aeromonas salmonicida *subsp. *smithia *and *Haemophilus **piscium *reveal pronounced similarities with *A. salmonicida *subsp. *salmonicida* Dis Aquat Org 1999 35 155 158 10.3354/dao035155 10092980

[B125] McCarthy DH Some ecological aspects of the bacterial fish pathogen - *Aeromonas salmonicida* Aquatic Microbiology Symp Soc Appl Bacteriol 1980 6 299 324

[B126] Belland RJ Trust TJ DNA:DNA reassociation analysis of *Aeromonas salmonicida* J Gen Microbiol 1988 134 307 315 3171541 10.1099/00221287-134-2-307

[B127] Eddy BP Cephalotrichous, fermentative Gram-negative bacteria: the genus *Aeromonas* J Appl Bacteriol 1960 23 216 248

[B128] Kluyver AJ Van Niel CB Prospects for a natural system of classification of bacteria Z Bakteriol Parasiten, Infekt Hyg, Orig 1936 94 369 403 2 Abt

[B129] Cowan ST Cowan and Steel's Manual for the Identification of Medical Bacteria 1974 2 Cambridge University Press, Cambridge

[B130] Kimura T A new subspecies of *Aeromonas salmonicida *as an etiological agent of furunculosis on "Sakuramasu" *(Oncorhynchus masou) *and pink salmon *(O. gorbuscha) *rearing for maturity. Part 2. On the morphological and physiological properties Fish Pathol 1969 3 45 52

[B131] Liu PV Observations on the specificities of extra-cellular antigens of the genera *Aeromonas *and *Serratia* J Gen Microbiol 1961 24 145 153 13762795 10.1099/00221287-24-1-145

[B132] Popoff M Étude sur les *Aeromonas salmonicida*. II. Caracterisation des bactériophages actifs sur les "*Aeromonas salmonicida*" et lysotypie Ann Recherch Vét 1971 2 33 45

[B133] Popoff M Intérêt diagnostique d'un bactériophage spécifique des *Aeromonas salmonicida* Ann Rech Vét 1971 2 137 139

[B134] Kilian M A taxonomic study of the genus *Haemophilus*, with the proposal of a new species J Gen Microbiol 1976 93 9 62 772168 10.1099/00221287-93-1-9

[B135] Broom AK Sneath PHA Numerical taxonomy of *Haemophilus* J Gen Microbiol 1981 126 123 149 7334332 10.1099/00221287-126-1-123

[B136] Trust TJ Ishiguro EE Atkinson HM Relationship between *Haemophilus piscium *and *Aeromonas salmonicida *revealed by *Aeromonas hydrophila *bacteriophage FEMS Microbiol Lett 1980 9 199 201 10.1111/j.1574-6968.1980.tb05636.x

[B137] Dalsgaard I Gudmundsdóttir BK Helgason S Høie S Thoresen OF Wichardt UP Wiklund T Identification of atypical *Aeromonas salmonicida*: inter-laboratory evaluation and harmonization of methods J Appl Microbiol 1998 84 999 1006 10.1046/j.1365-2672.1998.00435.x 9717284

[B138] Ross AJ Rucker RR Ewing WH Description of a bacterium associated with redmouth disease of rainbow trout (*Salmo gairdneri*) Can J Microbiol 1966 12 763 70 10.1139/m66-103 6007992

[B139] Steinum T Sjastad K Falk K Kvellestad A Colquhuin DJ An RT PCR-DGGE survey of gill-associated bacteria in Norwegian seawater-reared Atlantic salmon suffering proliferative gill inflammation Aquaculture 2009 293 172 179 10.1016/j.aquaculture.2009.05.006

[B140] Ewing WH Ross AJ Brenner DJ Fanning GR *Yersinia ruckeri *sp. nov., the redmouth (RM) bacterium Int J Syst Bacteriol 1978 28 37 44 10.1099/00207713-28-1-37

[B141] Steigerwalt AG Fanning GR Fife-Asbury MA Brenner DJ DNA relatedness among species of *Enterobacter *and *Serratia* Can J Microbiol 1976 22 121 137 10.1139/m76-018 1260520

[B142] Bercovier H Mollaret HH Krieg NR, Holt JG Genus XIV. *Yersinia *van Loghem 1944, 15AL Bergey's Manual of Systematic Bacteriology 1984 I Williams and Wilkins, Baltimore 489 506

[B143] Austin DA Robertson PAW Austin B Recovery of a new biogroup of *Yersinia ruckeri *from diseased rainbow trout *(Oncorhynchus mykiss*, Walbaum) Syst Appl Microbiol 2005 26 127 131 10.1078/072320203322337416 12747420

[B144] Fouz B Zarza C Amaro C First description of non-motile *Yersinia ruckeri *serovar I strains causing disease in rainbow trout, *Oncorhynchus mykiss *(Walbaum), cultured in Spain J Fish Dis 2006 29 339 346 10.1111/j.1365-2761.2006.00723.x 16768714

[B145] Smith IW A disease of finnock due to *Vibrio anguillarum* J Gen Microbiol 1961 74 247 252

[B146] Kaper JB Lockman H Remmers EF Kristensen K Colwell RR Numerical taxonomy of vibrios isolated from estuarine environments Int J Syst Bacteriol 1983 33 229 255 10.1099/00207713-33-2-229

[B147] Pazos F Santos Y Magariños B Bandín I Núñez S Toranzo AE Phenotypic characteristics and virulence of *Vibrio anguillarum - *related organisms Appl Environ Microbiol 1993 59 2969 2976 16349042 10.1128/aem.59.9.2969-2976.1993 PMC182394

[B148] Olsen JE Larsen JL Ribotypes and plasmid content of *Vibrio anguillarum *strains in relation to serovar Appl Environ Microbiol 1993 59 3863 3870 7506897 10.1128/aem.59.11.3863-3870.1993 PMC182542

[B149] Austin B Alsina M Austin DA Blanch AR Grimont F Grimont PAD Jofre J Koblavi S Larsen JL Pedersen K Tiainen T Verdonck L Swings J Identification and typing of *Vibrio anguillarum *a comparison of different methods Syst Appl Microbiol 1995 18 285 302

[B150] Austin B Austin DA Blanch AR Cerdà M Grimont F Grimont PAD Jofre J Koblavi S Larsen JL Pedersen K Tiainen T Verdonck L Swings J A comparison of methods for the typing of fish-pathogenic *Vibrio *spp Syst Appl Microbiol 1997 20 89 101

[B151] Schiewe MH Taxonomic status of marine vibrios pathogenic for salmonid fish Dev Biol Stand 1981 49 149 158

[B152] Muthiara LW Raymond BT Dekievit TR Stevenson RHW Antibody specificities of polyclonal rabbit and rainbow trout antisera against *Vibrio ordalii *and serotype O:2 strains of *Vibrio anguillarum* Can J Microbiol 1993 39 492 499 10.1139/m93-070 7687195

[B153] Sørensen UBS Larsen JL Serotyping of *Vibrio anguillarum* Appl Environ Microbiol 1986 51 593 597 3963811 10.1128/aem.51.3.593-597.1986 PMC238924

[B154] Grisez L Ollevier F Comparative serology of the marine fish pathogen *Vibrio anguillarum* Appl Environ Microbiol 1995 61 4367 4373 16535190 10.1128/aem.61.12.4367-4373.1995 PMC1388655

[B155] Pedersen K Dalsgaard I Larsen JL Characterization of atypical *Aeromonas salmonicida *isolates by ribotyping and plasmid profiling J Appl Bacteriol 1996 80 37 44 8698652 10.1111/j.1365-2672.1996.tb03187.x

[B156] Pedersen K Tiainen T Larsen JL Plasmid profiles, restriction length polymorphisms and O-serotypes among *Vibrio anguillarum *isolates Epidemiol Infect 1996 117 471 478 10.1017/S0950268800059136 8972671 PMC2271657

[B157] Kitao T Aoki T Fukodome M Kawano K Wada Y Mizuno Y Serotyping of *Vibrio anguillarum *isolated from diseased freshwater fish in Japan J Fish Dis 1983 6 175 181 10.1111/j.1365-2761.1983.tb00064.x

[B158] Tiainen T Pedersen K Larsen JL Immunological reactivity of *Vibrio anguillarum *sero-subgroups O2a and O2b, and comparison of their lipopolysaccharide profiles Curr Microbiol 1997 34 38 42 10.1007/s002849900141 8939800

[B159] O'Hici B Olivier G Powell R Genetic diversity of the fish pathogen *Aeromonas salmonicida *demonstrated by random amplified polymorphic DNA and pulsed-field gel electrophoresis analyses Dis Aquat Org 2000 39 109 119 10715816 10.3354/dao039109

[B160] Umelo E Trust TJ Identification and molecular characterization of two tandomly located flagellin genes from *Aeromonas salmonicida *A449 J Bacteriol 1997 179 5292 5299 9286979 10.1128/jb.179.17.5292-5299.1997 PMC179395

[B161] McIntosh D Austin B Atypical characteristics of the salmonid pathogen *Aeromonas salmonicida* J Gen Microbiol 1991 137 1341 1343 1919508 10.1099/00221287-137-6-1341

